# Vector competence of selected North American Culex and Coquillettidia mosquitoes for West Nile virus.

**DOI:** 10.3201/eid0706.010617

**Published:** 2001

**Authors:** M R Sardelis, M J Turell, D J Dohm, M L O'Guinn

**Affiliations:** Division of Tropical Public Health, Uniformed Services University of the Health Sciences, Bethesda, Maryland, USA.

## Abstract

To control West Nile virus (WNV), it is necessary to know which mosquitoes are able to transmit this virus. Therefore, we evaluated the WNV vector potential of several North American mosquito species. Culex restuans and Cx. salinarius, two species from which WNV was isolated in New York in 2000, were efficient laboratory vectors. Cx. quinquefasciatus and Cx. nigripalpus from Florida were competent but only moderately efficient vectors. Coquillettidia perturbans was an inefficient laboratory vector. As WNV extends its range, exposure of additional mosquito species may alter its epidemiology.


### Vector Competence of Selected North American *Culex* and *Coquillettidia* Mosquitoes for *West Nile Virus*

**Michael R. Sardelis,* Michael J. Turell,† David J. Dohm,† and Monica L. O'Guinn†**
*Uniformed Services University of the Health Sciences, Bethesda, Maryland, USA; and †U.S. Army Medical Research Institute of Infectious Diseases, Fort Detrick, Maryland, USA

To control *West Nile virus* (WNV), it is necessary to know which mosquitoes are able to transmit this virus. Therefore, we evaluated the WNV vector potential of several North American mosquito species. *Culex restuans* and *Cx. salinarius,* two species from which WNV was isolated in New York in 2000, were efficient laboratory vectors. *Cx. quinquefasciatus* and *Cx. nigripalpus* from Florida were competent but only moderately efficient vectors. *Coquillettidia perturbans* was an inefficient laboratory vector. As WNV extends its range, exposure of additional mosquito species may alter its epidemiology.

In 1999, *West Nile virus* (WNV) was recognized for the first time in the Western Hemisphere, causing human, equine, and avian deaths (1-4). Entomologic investigations of this outbreak resulted in the isolation of WNV from two mosquito species, *Aedes vexans* and *Culex pipiens* (2). The distribution of WNV in the United States expanded in 2000 from four northeastern states (Connecticut, Maryland, New Jersey, and New York) to eight additional eastern states (Delaware, Massachusetts, New Hampshire, North Carolina, Pennsylvania, Rhode Island, Vermont, and Virginia) and the District of Columbia (4).

During 2000, evidence of WNV infection was reported in nine additional mosquito species (4). These isolation studies provide preliminary evidence of involvement of several mosquito species in the transmission cycle. However, it is necessary to determine if any of these species are able to transmit WNV by bite before they can be implicated as vectors. In addition, the population density, host preference, feeding behavior, longevity, and seasonal activity of each mosquito species must be considered in determining its relative importance.

In Africa, southern Europe, and western Asia, WNV has been enzootic for many years, with isolations from >40 mosquito species, most in the genus *Culex* (5,6). Laboratory studies indicate that many *Culex* and *Aedes* species in the traditional enzootic range of WNV are competent laboratory vectors (5,6). However, because the introduction of WNV to the United States was recent, little is known about the potential for North American mosquito species to act as vectors of this virus.

Preliminary studies with North American mosquitoes indicate that New York strains of *Cx. pipiens* and *Ae. vexans* are competent but only moderately efficient laboratory vectors (7). The vector competence of *Ae. aegypti, Ae. albopictus, Ochlerotatus atropalpus, Oc. j. japonicus, Oc. sollicitans,* and *Oc. taeniorhynchus* for WNV has since been evaluated (8,9). WNV was isolated from *Cx. restuans* and *Cx. salinarius* caught during the 2000 outbreak in New York (4); however, the ability of these species to transmit WNV by bite is unknown. Other viruses circulating in the eastern United States have a similar epidemiology (e.g., *St. Louis encephalitis* [SLE] and *eastern equine encephalomyelitis* [EEE] viruses): they are maintained in an enzootic cycle involving birds as amplifying hosts and ornithophilic mosquitoes as enzootic vectors. Based on their association with these other arboviruses, several mosquito species should be considered potential vectors of WNV, although it has not yet spread to areas where these mosquitoes are found.

To assist public health personnel in assessing the risk that a potential mosquito vector represents for transmission of WNV, we conducted laboratory studies to evaluate the vector competence of *Cx. nigripalpus, Cx. quinquefasciatus, Cx. restuans, Cx. salinarius,* and *Coquillettidia perturbans.*

### Materials and Methods

### Mosquitoes

We tested five mosquito species for susceptibility to WNV (Table 1). *Cx. nigripalpus* was tested because it is the primary vector of SLE virus in Florida (10,11). *Cq. perturbans* is a potential epizootic vector of EEE virus in the eastern United States (12). *Cx. salinarius* has been found naturally infected with WNV (4) and has been implicated as a potential epizootic vector of EEE virus (12). *Cx. quinquefasciatus* has been implicated as a potential enzootic and epizootic vector of SLE virus (13). *Cx. restuans* has been found naturally infected with WNV (4) and may play a secondary role in the transmission and maintenance of SLE virus (14).

Address for correspondence: Michael J. Turell, Department of Vector Assessment, Virology Division, USAMRIID, 1425 Porter Street, Fort Detrick, MD 21702-5011, USA; fax: 301-619-2290; e-mail: michael.turell@det.amedd.army.mil

Table 1. Mosquito species tested for susceptibility to infection with *West Nile virus*

| *Species* | Strain | Source (year collected) | Generation |
|---|---|---|---|
| *Culex nigripalpus* | Indian River | Indian River, FL (2000) | $F_{0-1}$ |
| *Cx. quinquefasciatus* | Sebring | Sebring County, FL (1988) | $>F_{30}$ |
| *Cx. quinquefasciatus* | Vero Beach | Vero Beach, FL (1999) | $F_{10-12}$ |
| *Cx. restuans* | Maryland | Frederick & Prince George's Counties, MD (2000,2001) | $F_0$ |
| *Cx. salinarius* | Chambers | Chambers Co., TX (1992) | $>F_{30}$ |
| *Cq. perturbans* | Laurel | Laurel, MD (2000) | $F_0$ |

### Virus and Virus Assay

The WNV strain (Crow 397-99) used was isolated from a dead crow found in the Bronx, New York, during an epizootic in 1999 (7); it had been passaged once in Vero cell culture. Stocks of virus at a concentration of $10^{4.2}$ PFU/mL were prepared in a standard diluent (10% heat-inactivated fetal bovine serum in Medium 199 with Earle's salts [GIBCO-BRL, Gaithersburg, MD] $NaHCO_3$, and antibiotics). Viral stocks, triturated mosquito suspensions, and chicken blood samples were tested for infectious virus by plaque assay on Vero cells as described (15), except that the second overlay, containing neutral red stain, was added 2 days after the first overlay.

### Vector Competence Studies

Mosquitoes were allowed to feed on 2- to 3-day-old leghorn chickens (*Gallus gallus*) that had been inoculated with approximately $10^3$ PFU of WNV 1 to 2 days earlier. Immediately after the mosquitoes fed, blood was drawn from the jugular vein of each chicken (0.1 mL of blood into 0.9 mL of heparinized diluent), and the blood suspensions were frozen at –70°C until assayed for virus to determine viremias at the time of mosquito feeding. After feeding on viremic chickens, engorged mosquitoes were transferred to 3.8-L screen-topped cardboard cages and held at 26°C with a 16:8(L:D)-hour photoperiod. After an incubation period of 12 to 14 days, the mosquitoes were allowed to feed again on 1- to 2-day-old chickens, either individually or in small groups, to determine if they could transmit virus by bite. Immediately after the transmission attempt, the mosquitoes were killed by freezing, their feeding status was determined, and their legs and bodies were triturated separately in 1 mL of diluent.

Infection was determined by recovery of virus from the mosquito tissue suspension. If virus was recovered from its body but not its legs, the mosquito was considered to have a nondisseminated infection limited to its midgut. If virus was recovered from both the body and leg suspensions, the mosquito was considered to have a disseminated infection (16). We defined the infection and dissemination rates as the percentages of mosquitoes tested that contained virus in their body or legs, respectively. Chickens used in the transmission attempts were bled from the jugular vein 2 days after mosquito feeding, and the blood was handled as described above. Recovery of virus from this blood indicated transmission (9).

To examine viral transmission more efficiently, some of the unfed mosquitoes were inoculated intrathoracically (17) with 0.3 µL of a viral suspension containing $10^{4.2}$ PFU of WNV/mL ($10^{0.7}$ PFU/mosquito), held 7 to 14 days, and allowed to feed on 1- to 2-day-old chickens. Mosquitoes and blood specimens from these chicks were processed as described for the orally exposed mosquitoes.

To estimate transmission rates by species, we determined the percentage of mosquitoes with disseminated infection (after either oral exposure or by intrathoracic inoculation) that transmitted virus by bite. We then multiplied that percentage times the percentage of mosquitoes that developed a disseminated infection after feeding on a host with a particular viremia. The result is the estimated transmission rate for those mosquitoes.

### Statistical Analysis

Confidence intervals (95%) for infection and dissemination rates were calculated by SAS 8.0 (18). We used Fisher exact test to compare transmission rates among disseminated mosquitoes in each species. Significance was tested at a level of alpha = 0.05.

### Results

All mosquito species examined in this study were susceptible to infection with WNV and developed disseminated infections (Table 2). Infection rates were >84% in all the *Culex* species when the viral titer in the donor chicken was $\geq 10^{6.3}$ PFU/mL of blood. In contrast, the infection rate was 18% in *Cq. perturbans* fed on a chicken with a similar level of viremia. For most mosquito species tested, dissemination rates were approximately one fourth the infection rates.

None of the *Culex* species tested differed significantly in the percentages of mosquitoes with disseminated infection that transmitted virus (Table 3). However, the percentage of *Cq. perturbans* with disseminated infection that transmitted WNV was significantly lower than that for *Cx. nigripalpus* and *Cx. quinquefasciatus* (Fisher exact test, $p < 0.01$).

We used the percentage of mosquitoes with disseminated infection that transmitted virus from Table 3 and the dissemination rates at 14 days after the infectious blood meal from Table 2 to estimate the transmission rate for each species. Under laboratory conditions and at the highest viral dose tested, the *Culex* species tested were moderately efficient vectors (estimated transmission rates 10% to 55%). In contrast, *Cq. perturbans* was an inefficient vector (estimated transmission rate $\leq$2%) (Table 2).

### Conclusions

Previous laboratory studies indicate that a number of North American mosquito species could serve as vectors of WNV (7-9). Our study indicated that several additional *Culex* species and *Cq. perturbans* are potential vectors of WNV. The viremias used in our study, $10^{5.5-7.5}$ PFU/mL of blood, are consistent with levels considered to be low to moderate viremias for hooded crows and house sparrows in Egypt (19) and experimentally infected North American house sparrows and other passerine birds (N. Komar, pers.

Table 2. Infection, dissemination and estimated transmission rates for mosquitoes orally exposed to *West Nile virus*

| Species | Strain | Viral dose[a] | No. tested | Infection rate[b] | Dissemination rate[c] | Estimated transmission rate[d] |
|---|---|---|---|---|---|---|
| *Culex nigripalpus* | Indian River | 4.6 | 7 | 29 ([4-71], 2) | 0 ([0-41], 0) | 0 |
| | Indian River | 5.7±0.5 | 132 | 78 ([70-85], 103) | 8 ([4-14], 11) | 7 |
| | Indian River | 6.8±0.4 | 127 | 84 ([77-90], 107) | 12 ([7-19], 15) | 10 |
| *Cx. quinquefasciatus* | Sebring | 5.5 | 16 | 50 ([25-75], 8) | 6 ([0-30], 1) | 6 |
| | Sebring | 7.0±0.5 | 78 | 91 ([82-96], 71) | 22 ([13-33], 17) | 20 |
| | Vero Beach | 5.0 | 13 | 46 ([19-75], 6) | 0 ([0-25], 0) | 0 |
| | Vero Beach | 6.3 | 17 | 94 ([71-100], 16) | 12 ([1-36], 2) | $\leq$13 |
| *Cx. restuans* | Maryland | 6.6±0.3 | 11 | 100 ([72-100], 11) | 55 ([23-83], 6) | 55 |
| *Cx. salinarius* | Chambers | 6.6±0.3 | 20 | 95 ([75-100], 19) | 60 ([36-81], 12) | 34 |
| *Coquillettidia perturbans* | Laurel | 6.6±0.3 | 11 | 18 ([2-52], 2) | 9 ([0-41], 1) | 2 |

[a]$Log_{10}$ PFU/mL of blood.
[b]Percentage of mosquitoes containing virus in their bodies ([95% confidence interval (CI)], number infected).
[c]Percentage of mosquitoes containing virus in their legs ([95% CI], number disseminated).
[d]The estimated transmission rate = the percentage of mosquitoes that developed disseminated infection 12-14 days after ingesting WNV multiplied by the percentage of mosquitoes with disseminated infection that transmitted virus by bite (Table 3).

comm.). Thus, our results should reflect what would happen when mosquitoes feed on birds circulating a similar concentration of virus in nature.

The *Culex* species tested in this study were moderately efficient vectors, with estimated transmission rates from 10% to 55% after exposure to viremias $\geq 10^{6.3}$. For comparison, the estimated transmission rate for *Cx. pipiens* held under conditions similar to those of our study is 20% (9). Although the *Culex* species tested were readily susceptible to oral infection, most infections were limited to the midgut and did not disseminate to the hemocoel. This finding is similar to results reported for *Cx. pipiens*, in which 81% became infected but only 23% developed disseminated infection (9). Compared with the moderately efficient *Culex* mosquito vectors of WNV, selected container-breeding *Aedes* and *Ochlerotatus* species are highly efficient vectors, and selected floodwater *Aedes* and *Ochlerotatus* mosquitoes are inefficient laboratory vectors (7-9). The *Cq. perturbans* in our study fell into the inefficient vector category.

*Cx. nigripalpus* has not been found naturally infected with WNV. However, the distribution of WNV in the United States is just beginning to reach the southern half of North Carolina, the northernmost limit of these mosquitoes' geographic distribution. *Cx. nigripalpus* is likely to become involved in WNV transmission because it is a primary vector of SLE in Florida (10,11) and is a competent laboratory vector of WNV. Furthermore, *Cx. nigripalpus* is an opportunistic feeder (20,21) and shifts host selection based on the season, feeding on avian hosts in the winter and spring and on mammalian hosts in the summer and fall (22). These factors, coupled with the vector competence data, suggest that *Cx. nigripalpus* could serve as an epizootic as well as an enzootic vector for WNV.

Our study showed that *Cx. quinquefasciatus* can transmit WNV by bite. WNV has not been isolated from wild-caught *Cx. quinquefasciatus*. However, the current distribution of WNV is just beginning to overlap the geographic range of this species (generally the southern United States). *Cx. quinquefasciatus* has been implicated (through virus isolation and abundance during outbreaks) in the rural transmission of SLE virus in the western United States (23) and in urban transmission of SLE virus in the southern United States (24). In contrast to *Cx. pipiens*, which primarily feeds on birds, *Cx. quinquefasciatus* shows a preference for avian blood but will feed readily on mammals, including humans (25). The data from this study, the bionomics of *Cx. quinquefasciatus*, and the mosquitoes' association with an arbovirus with similar epidemiology to WNV, suggest that *Cx. quinquefasciatus* may play a role in WNV transmission if—or more likely when—the distribution of the mosquito and the virus overlap to a sufficient degree.

*Cx. restuans*, which has been found naturally infected with WNV (4), transmitted WNV by bite in our study. Similarly, this species has been implicated as a vector of SLE

Table 3. Percent of mosquitoes with disseminated infection (after either oral exposure to or intrathoracic inoculation with *West Nile virus*) that transmitted virus by bite

| Species (strain) | No. tested | Percent transmission[a] |
|---|---|---|
| *Culex nigripalpus* (Indian River) | 15 | 87 ([60-98],13)a |
| *Cx. quinquefasciatus* (Sebring) | 18 | 94 ([73-100],17)a |
| *Cx. quinquefasciatus* (Vero Beach) | Not determined | |
| *Cx. restuans* (Maryland) | 2 | 100 ([16-100],2) a,b |
| *Cx. salinarius* (Chambers) | 16 | 56 ([30-80],9)a,b |
| *Coquillettidia perturbans* (Laurel) | 17 | 24 ([7-50],4)b |

[a]Percentage of mosquitoes with disseminated infection that transmitted virus by bite (95% confidence interval), number transmitting). Percent transmissions followed by the same letter are not significantly different at alpha = 0.05 by Fisher exact test.

virus by virus isolations from field-collected specimens (26,27), and its role is supported by laboratory transmission studies (13). *Cx. restuans* breeds in ground pools or container habitats, is widespread in its distribution in the United States, and adults are active early (by mid-May) in the eastern United States (28). This early season abundance, along with coinciding isolations of SLE virus from this species in early summer, implies that it may be involved in the overwintering or amplification of SLE virus (26). The isolation of WNV from *Cx. restuans* in July in Connecticut (29), relatively early in the WNV transmission season, raises concern that the role of *Cx. restuans* in WNV transmission may be similar to the one suggested for SLE virus. *Cx. restuans* feeds primarily on avian hosts (30), but whether it feeds on humans remains unclear (31). Given the lack of firm data on host preference, the role of this species as an enzootic or epizootic vector of WNV is still uncertain.

Our study indicated that *Cx. salinarius* transmits WNV efficiently by bite. During 2000, evidence of WNV infection was reported in 35 pools of this species, second in number only to the number of positive pools (126) of *Cx. pipiens* (4). To date, no summary of the data (e.g., minimum infection rates) from the 2000 season has been published, so the relative importance of these isolates cannot be compared. In general, *Cx. salinarius* appears to be mammalophagic in studies of blood meals, but its host feeding pattern is thought to be opportunistic, depending on host availability, innate host preference, or combination of these factors (20,25,32,33). Given the number of WNV-positive pools, its vector competence for WNV, and its feeding behavior, *Cx. salinarius* may be an ideal bridge vector between the enzootic avian cycle of WN and mammalian hosts.

*Cq. perturbans* was the least efficient WNV vector of those we tested. Contributing heavily to this finding was the presence of a salivary gland barrier. Less than one fourth of *Cq. perturbans* with disseminated infection transmitted WNV by bite (Table 3). Furthermore, this is the only North American species tested so far that exhibits a substantial salivary gland barrier. *Cq. perturbans* is generally regarded as mammalophagic (30,34); however, there are reports of its feeding on wading birds and passerines (34-36) and of numerous EEE virus isolates from field-collected specimens (37-40). Despite the low transmission rate, the role of *Cq. perturbans* as a potential epizootic vector of WNV should not be totally discounted.

Our study extended the list of potential North American mosquito vectors of WNV. None of the North American species tested in this study or others (7-9) was refractory to WNV. However, there is a wide range in vector competence in these species, ranging from nearly incompetent (e.g., *Cq. perturbans*) to highly efficient (e.g., *Oc. j. japonicus*). These data are similar to those for Old World mosquito vectors of WNV, in which all *Aedes* and *Culex* species tested were competent vectors (5,6). Vector competence studies indicate that North American mosquitoes fall into three general categories depending on genera and, in some instances, breeding habitat: highly efficient, container-breeding *Aedes* and *Ochlerotatus* species; moderately efficient, *Culex* species; and inefficient, floodwater-breeding *Aedes* and *Ochlerotatus* and *Cq. perturbans*.

As WNV extends its range southward and westward, additional mosquito species (e.g., *Cx. nigripalpus*, *Cx. quinquefasciatus*, *Cx. tarsalis*, and *Ae. albopictus*) will have greater exposure to this virus. Involvement of some of the species, particularly container-breeding *Aedes* and *Ochlerotatus*, may alter the epidemiology of WNV and present additional control problems for mosquito abatement personnel. In addition, mosquitoes are more efficient vectors at warmer temperatures (41,42; Dohm, unpub. data), a factor that will further change the epidemiology of WNV as its range extends southward.

### Acknowledgments

We thank T. McNamara for providing the Crow 397-99 strain of WNV, D. Shroyer for providing *Cx. nigripalpus*, J. Nayar for providing the Vero Beach strain of *Cx. quinquefasciatus*, N. Achee for providing *Cq. perturbans*, and J. Olsen for providing the Sebring strain of *Cx. quinquefasciatus* and *Cx. salinarius*. We also thank J. Blow, R. Leon, and K. Kenyon for critically reading the manuscript.

In conducting research using animals, the investigators adhered to the "Guide for the Care and Use of Laboratory Animals," prepared by the Committee on Care and Use of Laboratory Animals of the Institute of Laboratory Animal Resources, National Research Council (NIH Pub. No. 86-23, Revised 1996). The facilities are fully accredited by the Association for Assessment and Accreditation of Laboratory Animal Care, International.

Major Sardelis is a graduate student in the Division of Tropical Public Health at the Uniformed Services University of Health Sciences. His research interests focus on the impact of newly invasive mosquito species on arbovirus transmission in the eastern United States and the distribution and bionomics of mosquitoes in the Amazon Basin region of Peru.

### References

1. Lanciotti RS, Roehrig JT, Deubel V, Smith J, Parker M, Steele K et al. Origin of the West Nile virus responsible for an outbreak of encephalitis in the Northeastern United States. Science 1999;286:2333-7.
2. Centers for Disease Control and Prevention. Outbreak of West Nile-like viral encephalitis—New York, 1999. MMWR Morb Mortal Wkly Rep 1999;48:845-9.
3. Centers for Disease Control and Prevention. Update: West Nile-like viral encephalitis—New York, 1999. MMWR Morb Mortal Wkly Rep 1999;48:890-2.
4. Centers for Disease Control and Prevention. Update: West Nile virus activity—Eastern United States, 2000. MMWR Morb Mortal Wkly Rep 2000;49:1044-7.
5. Hayes C. West Nile fever. In: Monath TP, editor. The Arboviruses: epidemiology and ecology, Vol V. Boca Raton (FL): CRC Press; 1989. p. 59-88.
6. Hubalek Z, Halouzka J. West Nile virus—a reemerging mosquito-borne viral disease in Europe. Emerg Infect Dis 1999;5:643-50.
7. Turell MJ, O'Guinn M, Oliver J. Potential for New York mosquitoes to transmit West Nile virus. Am J Trop Med Hyg 2000;62:413-4.
8. Sardelis MR, Turell MJ. *Ochlerotatus j. japonicus* in Frederick County, Maryland: discovery, distribution, and vector competence for West Nile virus. J Am Mosq Control Assoc 2001;17:137-41.
9. Turell MJ, O'Guinn ML, Dohm DJ, Jones JW. Vector competence of North American mosquitoes (Diptera: Culicidae) for West Nile virus. J Med Entomol 2001;38:130-4.
10. Day JF, Edman JD. Host location, blood-feeding, and oviposition behavior of *Culex nigripalpus* (Diptera: Culicidae): Their influence on St. Louis encephalitis virus transmission in southern Florida. In: Scott TW, Grumstup-Scott J, editors. Proceedings of a Symposium: The Role of Vector-Host Interactions in Disease Transmission. Misc Pubs Entomol Soc Am 1988;68:1-8.

11. Day JF, Stark LM. Frequency of Saint Louis encephalitis virus in humans from Florida, USA: 1990-1999. J Med Entomol 2000;37:626-33.
12. Scott TW, Weaver SC. Eastern equine encephalomyelitis virus: Epidemiology and evolution of mosquito transmission. Adv Virus Res 1989;37:277-328.
13. Chamberlain RW, Sudia WD, Gillett JD. St. Louis encephalitis virus in mosquitoes. Am J Hyg 1959;70:221-36.
14. Tsai TF, Mitchell CJ. St. Louis encephalitis. In: Monath TP, editor. The arboviruses: epidemiology and ecology. Vol IV. Boca Raton (FL): CRC Press; 1988. p. 113-43.
15. Gargan TP II, Bailey CL, Higbee GA, Gad A, El Said S. The effect of laboratory colonization on the vector pathogen interaction of Egyptian *Culex pipiens* and Rift Valley fever virus. Am J Trop Med Hyg 1983;32:1154-63.
16. Turell MJ, Gargan TP II, Bailey CL. Replication and dissemination of Rift Valley fever virus in *Culex pipiens*. Am J Trop Med Hyg 1984;33:176-81.
17. Rosen L, Gubler D. The use of mosquitoes to detect and propagate dengue viruses. Am J Trop Med Hyg 1974;23:1153-60.
18. SAS Institute Inc. SAS/STAT MULTTEST software release 8.00. Cary (NC): The Institute; 1999.
19. Work TH, Hurlbut HS, Taylor RM. Indigenous wild birds of the Nile Delta as potential West Nile virus circulating reservoirs. Am J Trop Med Hyg 1955;4:872-88.
20. Edman JD. Host-feeding patterns of Florida mosquitoes. III. *Culex* (*Culex*) and *Culex* (*Neoculex*). J Med Entomol 1974;11:95-104.
21. Nayar JK. Bionomics and physiology of *Culex nigripalpus* (Diptera: Culicidae) of Florida: An important vector of disease. Gainesville (FL): Florida Agricultural Experiment Station; 1982. Bull No. 827. 77 pp.
22. Edman JD, Taylor DJ. *Culex nigripalpus*: seasonal shift in the bird-mammal feeding ratio in a mosquito vector of human encephalitis. Science 1968;161:67-8.
23. Reisen WK, Meyer RP, Milby MM, Presser SB, Emmons RW, Hardy JL, et al. Ecological observations on the 1989 outbreak of St. Louis encephalitis virus in the southern San Joaquin Valley of California. J Med Entomol 1992;29:472-82.
24. Beadle LD, Menzies GC, Hayes GR Jr, Von Zuben FJ Jr, Eads RB. St. Louis encephalitis in Hidalgo County, Texas. Vector control and evaluation. Public Health Rep 1957;72:531.
25. Tempelis DH. Host-feeding patterns of mosquitoes, with a review of advances in analysis of blood meals by serology. J Med Entomol 1974;11:635-53.
26. Mitchell CJ, Francy DB, Monath TP. Arthropod vectors. In: Monath TP, editor. St. Louis encephalitis. Washington: American Public Health Association; 1980.
27. Monath TP. Arthropod-borne encephalitides in the Americas. Bull Wld Hlth Organ 1979;57:513-33.
28. Eldridge BF, Bailey CL, Johnson MD. A preliminary study of the seasonal geographic distribution and overwintering of *Culex restuans* Theobald and *Culex salinarius* Coquillett (Diptera: Culicidae). J Med Entomol 1972;9:233-8.
29. Centers for Disease Control and Prevention. Update: West Nile virus activity—Northeastern United States, January-August 7, 2000. MMWR Morb Mortal Wkly Rep 2000;49:714-7.
30. Horsfall WR. Mosquitoes, their bionomics and relation to disease. New York: Ronald Press; 1955: 723 pp.
31. Moore CG, McLean RG, Mitchell CJ, Nasci RS, Tsai TF, Calisher CH, et al. Guidelines for arbovirus surveillance programs in the United States. Fort Collins (CO): Centers for Disease Control and Prevention, U.S. Department of Health and Human Services; 1993. 83 pp.
32. Murphey FP, Burbutis PP, Bray DF. Bionomics of *Culex salinarius* Coquillett. II. Host acceptance and feeding by adult females of *C. salinarius* and other mosquito species. J Med Entomol 1967;11:739-48.
33. Cupp EW, Stokes GM. Feeding patterns of *Culex salinarius* Coquillett in Jefferson Parish, Louisiana. Mosq News 1973;36:332-5.
34. Edman JD. Host-feeding patterns of Florida mosquitoes. I. *Aedes, Anopheles, Coquillettidia, Mansonia* and *Psorophora*. J Med Entomol 1971;8:687-95.
35. Hayes RO. Host preference of *Culiseta melanura* and allied mosquitoes. Mosq News 1961;21:179-82.
36. Magnarelli LA. Host feeding patterns of Connecticut mosquitoes (Diptera: Culicidae). Am J Trop Med Hyg 1977;26:547-52.
37. Howitt NF, Dodge HR, Bishop LK, Gorrie RH. Recovery of the virus of eastern equine encephalomyelitis from mosquitoes (*Mansonia perturbans*) collected in Georgia. Science 1949;110:141-2.
38. Sudia WD, Chamberlain RW, Coleman PH. Arbovirus isolations from mosquitoes collected in South Alabama, 1959-1963, serologic evidence of human infection. Am J Epidemiol 1968;87:112-26.
39. Crans WJ, Schulze TL. Evidence incriminating *Coquillettidia perturbans* (Diptera: Culicidae) as an epizootic vector of eastern equine encephalitis. I. Isolation of EEE virus from *Cq. perturbans* during an epizootic among horses in New Jersey. Bull Soc Vector Ecol 1986;11:178-84.
40. Andreadis TG, Anderson JF, Tirrell-Peck SJ. Multiple isolations of eastern equine encephalitis and highlands J viruses from mosquitoes (Diptera: Culicidae) during a 1996 epizootic in southeastern Connecticut. J Med Entomol 1998;35:296-302.
41. Jupp PG. Laboratory Studies on the Transmission of West Nile by *Culex (Culex) univittatus* Theobald; factors influencing the transmission rate. J Med Entomol 1974;11:455-8.
42. Cornel AJ, Jupp PG, Blackburn NK. Environmental temperature on the vector competence of *Culex univittatus* (Diptera: Culicidae) for West Nile virus. J Med Entomol 1993;30:449-56.
